# Similarities and differences of graduate entry-level competencies of chiropractic councils on education: a systematic review

**DOI:** 10.1186/s12998-016-0084-0

**Published:** 2016-01-21

**Authors:** Stanley I. Innes, Charlotte Leboeuf-Yde, Bruce F. Walker

**Affiliations:** School of Health Professions, Murdoch University, Murdoch, Australia; Institut Franco-Européen de Chiropraxie, Ivry sur Seine, France; Complexité, Innovation et Activités Motrices et Sportives, UFR STAPS, Université Paris Sud-11, Orsay Cedex, France

**Keywords:** Councils of chiropractic education, Competence, Practice profiles, Standards of education, Similarities, Differences

## Abstract

**Background:**

Councils of Chiropractic Education (CCE) indirectly influence patient care and safety through their role of ensuring the standards of training delivered by chiropractic educational institutions. This is achieved by CCEs defining competence and creating lists of descriptive statements to establish the necessary standards for students to attain before graduating. A preliminary review suggested that these definitions and descriptive lists lacked consensus. This creates the potential for variations in standards between the CCE jurisdictions and may compromise patient care and safety and also inter-jurisdictional mutual recognition. The purposes of this study were 1) to investigate similarities and differences between the CCEs in their definitions of competence, domains of educational competencies, components of the domains of competencies, as represented by assessment and diagnosis, ethics, intellectual development, and 2) to make recommendations, if significant deficiencies were found.

**Method:**

We undertook a systematic review of the similarities and differences between various CCEs definitions of competence and the descriptive lists of educational competencies they have adopted. CCEs were selected on the basis of WHO recommendations. Blinded investigators selected the data from CCE websites and direct contact with CCEs. This information was tabulated for a comparative analysis.

**Results:**

All CCEs’ definitions of competence included the elements of “knowledge”, “skills” and “attitudes” whereas only one CCE included the expected “abilities” element. The educational application of the definition of competency among CCEs varied. A high level of similarity when comparing the domains of competence adopted by CCEs was found despite variations in the structure.

Differences between CCEs became increasingly apparent when the three selected representative domains were compared. CCEs were found to stipulate varying levels of prescriptiveness for graduate entry level standards.

**Conclusions:**

A series of recommendations are proposed to create uniform and high quality international standards of care. Future research should compare the levels of CCEs enforcement of standards to see if similarities and differences exist.

## Introduction

Chiropractors are trained worldwide in different types of institutions; most are private colleges but some are integrated into state funded universities. Accreditation authorities ensure that there are professional standards that must be met in chiropractic pre-professional training so that patients are protected and treated properly by graduates from those programs. These accreditation authorities are usually empowered or accredited to do this by their respective governments. In this way individual colleges do not have full power to determine their own course criteria. For chiropractic educational institution standards this control mechanism of course accreditation is carried out by various Chiropractic Councils of Education (CCE). These CCEs are located in North America, Australia, Canada, and Europe. There is also an international umbrella council of chiropractic education organization known as the Chiropractic Council of Education International (CCE-Int) [1]. The World Health Organization (WHO) recommends the CCE-Int as the consultative body for national health authorities when evaluating chiropractic training programs [2].

Educational standards of the various institutions are defined and monitored by the CCEs which enforce this by inspecting and evaluating the chiropractic institutions’ facilities and educational programs. CCEs achieve this, in part, by defining competence and creating lists of descriptive statements to clarify the necessary knowledge, understanding, skills, attitudes, and behaviours students should attain before graduating and entering practice [3]. These competencies are an important means by which regulatory bodies can change professional standards of practice [4].

### Defining Competency

The conceptualisation of competence has important implications for the way that competence based medical education is implemented [5]. The Oxford Concise Dictionary defines competence as “the ability to do something successfully or efficiently” [6]. However, it has been suggested that one broad definition is not suitable for all professions [7] and what is required are specific definitions and competencies that have sufficient detail and clarity to be professionally useful [8].

Efforts to make the use of competencies more profession specific and effective increased in the 1960s as companies sought to assess an individual’s expected performance levels, skills and knowledge [9]. Today competencies are extensively applied to describe expectations of graduating students in medical and allied health professions [3]. This level of specificity is required to detail the domains for the practitioner to function successfully within that discipline. For this reason it is appropriate for CCEs to define and prescribe these domains and their components which are required to produce competent chiropractic clinicians. This process should result in a high standard of chiropractic education at internationally comparable levels [10].

There is not a universally agreed conceptualisation of competence in medical education. A systematic review of medical definitions of competence, concluded that the words “knowledge, skills and other” were the most commonly occurring components [5] The authors allocated all other words to a general category “other components”. Here, “attitudes” and “abilities” were prevalent and also suggested as essential ingredients of competence. “Skills” were defined as being related to manual dexterity while “ability” seen to be was commonly composed of abstract reasoning, memory and the cognitive processes associated with solving novel questions.

### Problem

There is evidence of variations in practitioner profiles [11] as seen in a recent study of Canadian chiropractors that showed differences in vaccination beliefs, X-ray usage, referral patterns, and treatment types, some of which must be considered unsuitable. These unsuitable profiles were found to be stemming from a cluster of accredited educational institutions in North America [12]. Another Canadian study found a relationship between the accredited educational institution of graduation and chiropractors’ interactions with other health professionals, as measured by receiving patient referrals from medical doctors [13]. The chiropractors less likely to receive referrals were more likely to take their own radiographs, treat a higher percentage of patients for somatovisceral conditions and consider maintenance/wellness care as a main component of practice activity. These findings support the possibility that there are differing standards of CCE requirements resulting in differing graduate outcomes.

This may be the case because laws and scope of practice may differ on a country-by-country basis. Thus, a CCE for a regional part of the world may reflect those differences as a result of scope. Another possible explanation, which may account in part for these differences are differing standards of the various CCEs because of contextual independence and in selections of definitions of competence. This may result in differences in standards between jurisdictions perhaps resulting in dissimilarities in practitioner profiles. There is evidence that this is the case for medicine [14, 15].

Some practice profiles are clearly undesirable. For example, information obtained from the Chiropractic Board of Australia [16], Wisconsin Chiropractic Examining Board [17], and previous research [18] suggests that the competency domains of 1) patient assessment and diagnosis, 2) ethics, and 3) intellectual and professional development are matters that commonly appear in registration or licensing board complaint investigations. Consequently, analysis of similarities and differences in these three key domains between all CCEs is important when looking for differences in standards that may result in desirable or undesirable practice profiles and uniformity of standards worldwide.

In summary, the literature confirms that competence is not uniformly conceptualised in health education. Similarities and differences exist between and within professions [5] and educational competencies used to describe high standards of practice, need to be profession specific. Variations between the definitions of the different CCEs and prescriptive lists describing competency may result in differing practitioner profiles, which may create differences in the quality of care and patient safety. Ultimately, an unequal standard and overly varied treatment approach may also impact on the international mobility of chiropractors.

### Aim

The aim of this systematic review was to investigate similarities and differences between the various CCEs in their definitions of graduate competency and the educational competencies they have adopted.

### Objectives

The objectives were to review: 1. CCE definitions of competence; 2. domains of educational competencies; 3. components of the domains of competencies, as represented by assessment and diagnosis, ethics, and intellectual development, and 4. to make recommendations, if significant deficiencies were found.

## Method

We conducted a systematic review to investigate the first three objectives. Protocols for clinical systematic reviews are recommended to be prospectively registered where possible (PRISMA [19, 20] PROSPERO [20]), However, as this systematic review focussed on the descriptive definitions in documents obtained from CCEs used for educational standards for chiropractic competence and not peer reviewed journal articles, it was not eligible for prospective registration with databases such as PROSPERO [21].

### Eligibility criteria

The WHO recommends the CCE-Int as the source of information regarding evaluation of chiropractic education [2]. Consequently, for CCE inclusion, we used this recommendation meaning that a CCE used in our study had to be recognized by the CCE-Int and be a member in good standing. The Council on Chiropractic Education (CCE-USA), Council on Chiropractic Education Australasia (CCE-Australia), European Council on Chiropractic Education (ECCE), and Council on Chiropractic Education Canada (CCE-Canada) all met the inclusion criteria.

### Data extraction process and synthesis of results

The respective CCE websites were identified and searched independently by the lead author and a research assistant. All CCEs were asked in writing whether additional relevant information was available that was not available on their respective websites.

A Masters in Business Administration graduate experienced with organisational evaluation acted as a research assistant and was instructed on the search domains. A training exercise was undertaken to establish a consistent process for extracting data from the websites. The research assistant was instructed on the aims and objectives of the project. Further, the roles of the CCEs were defined. The independent reviewer conducted a web search to locate the CCEs. The lead author and the research assistant then independently searched the CCE websites to identify and extract a definition of competence. The extracted data was recorded and tabulated. The author and research assistant then compared these for agreement. A third investigator was available to resolve any conflicts. The table format for the definitions was structured to identify similarities and differences with respect to the elements of “knowledge”, “skills”, “abilities”, “other components”, and “profession specific details”.

The same process was repeated for the extraction of competency lists for each CCE.

**Fig. 1:**
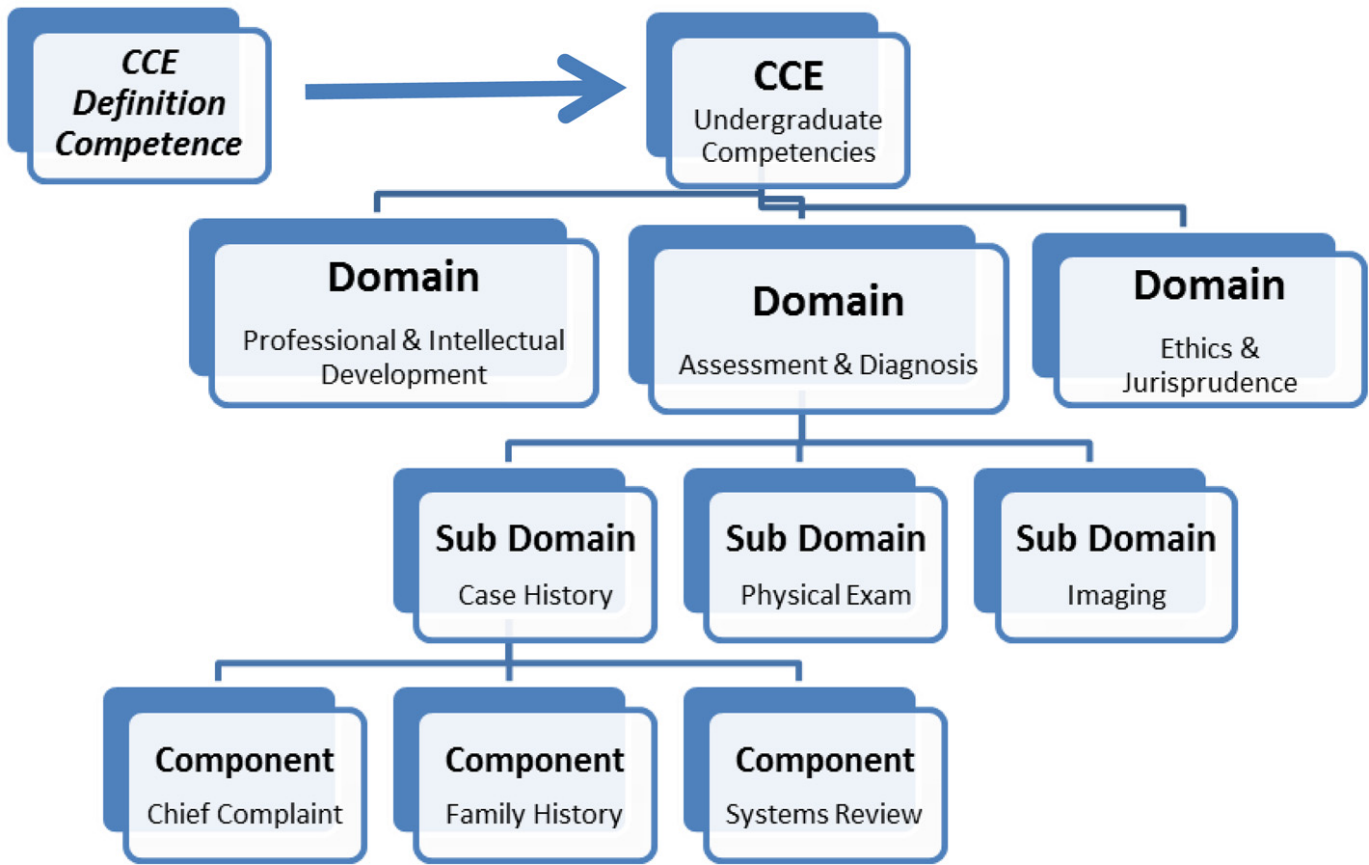
Illustrative diagram of structure of our systematic review

Finally, the components of the three selected domains (professional and intellectual development, assessment and diagnosis, ethics and jurisprudence,) were extracted and placed into a tabular format and analysed for similarities and differences (Fig. 1).

The CCE definition of competence informs the comprehension and construction of undergraduate competencies. Competence is deconstructed into a series of domains thought to describe chiropractic practice. Each of these domains is further deconstructed into smaller subdomains and finally components which are intended to be measureable behaviours and outcomes.

## Results

The research assistant and lead author (SI) extractions agreed on 4 of the 5 definitions of competence. After discussion consensus was reached on the one CCE definition mismatch and did not require independent adjudication. There was agreement between both researcher and research assistant on all 4 of the CCE lists of competency. This resulted in a match on 8 of the 9 data extractions.

The CCE-Int and the four regional CCEs (CCE-USA, CCE-Aust, CCE-Canada, ECCE) were included for the first objective of comparing competency definitions. The CCE-Int did not have any graduate entry-level standards for competency and could not be included in the analysis for the second and third objectives.

All CCEs responded to the request asking about the presence of additional information apart from their websites, and all stated that there was no additional information.

The investigators agreed on all definitions and competency selections from the respective CCE websites.

The ratios of CCE domains and components of domains were found to vary considerably. The largest was found for the CCE-Aust which had 11 domains with 299 components describing these domains (Table 1), resulting in a ratio of 27.2 components per domain. The smallest was noted for the ECCE with 3 domains and 21 components (i.e. a ratio of 7.0).

**Table 1:** Number of domains and component statements and ratios of these among the CCEs

	CCE-Aust	CCE-Canada	ECCE	CCE-USA
*Domains*	11	14	3	7
*Component statements*	299	213	21	63
*Component/Domain ratio*	27.2	15.2	7.0	9.0

### Objective 1: definitions of competency

All the CCEs used definitions of competence that included two of the three basic elements, namely *knowledge* and *skills.* Another word common to all five CCEs was *attributes* (See Table 2). Only the ECCE included the expected third element of *abilities* and this was used with respect to problem solving.

**Table 2:** Definitions of competency used by the major regulatory bodies

Name of CCE	Definition of “competency”	Knowledge	Skills	Attitudes	Context	Other
CCE-Int	the practice of chiropractic requires the acquisition of *relevant knowledge, understandings, attitudes, habits and psychomotor skills* (pg 3, 2010)	X	X	X	Practice of chiropractic	Habits
CCE-Aust	Competencies: Written statements describing the levels of knowledge, skills and attitudes expected of graduates (pg 18, 2009).	X	X	X	practitioner	
ECC-Europe	a measurable set of *skills, knowledge, problem solving abilities and attitudes* in controlled representations of professional practice when performing at maximum levels of ability (pg 57, 2013).	X	X	X	Professional practice	Problem solving abilities
CCE-Canada	a student’s *knowledge, skills and attitudes* with the goals of providing feedback to enhance the educational progress, rating performance, and determining the appropriateness of progression in the clinical phase of becoming a qualified chiropractor (pg 68, 2011).	X	X	X	Qualified chiropractor	
CCE (USA)	Mandatory meta-competencies have been identified regarding the *skills, attitudes, and knowledge* that a doctor of chiropractic program provides so that graduates will be prepared to serve as primary care chiropractic physicians (pg 21, 2013)	X	X	X	Chiropractic physician	
Aust. National Health Work Force	It refers to specific capabilities in applying particular *knowledge, skills, decision-making attributes and values* to perform tasks safely and effectively in a specific health workforce role (pg 5, 2011)	X	X		Health workforce role	Values, decision making attributes

Three CCEs included words from the “other components” category. First, the CCE-Int definition specified that the skills necessary for the competent practice of chiropractic are psychomotor in nature, and that these should become “*habits*”. Second, the ECCE added “*problem solving abilities and attitudes*”. Third, the CCE-USA used the term “*meta-competencies*”.

The CCEs did not have a common function or context for application of the definition of competency. The CCE-Canada described the use of competencies as a feedback mechanism for *“monitoring the educational progression toward becoming a chiropractor”.* The CCE-Aust and the CCE-USA definitions were used to determine if a student was ready to graduate and enter solo practice. The ECCE applied the definition to “*controlled representations of professional practice while performing at maximum levels of ability”.*

### Objective 2: domains of competency

There was inconsistency in structure among the CCEs for domains of competency and this affected the methodology for data extraction. For example, the CCE-USA had only 7 areas or domains but the CCE-Canada had the greatest number with 14. Consequently the CCE-Canada domains were chosen as the basis for the table structure of comparative purposes because it included all the available information found in the other CCEs and would therefore enable the identification of apparently absent domains. These 14 domains of competency were presented in Table 3.

**Table 3:** Comparison of common competency domains of CCEs

*Major elements/ domains of competency*	CCE-USA	CCE-Aust	ECC-Europe	CCE-Canada
History taking	X	X	X	X
Physical exam	X	X	X	X
Neuromusculoskeletal exam		X		X
Psychosocial assessment + cultural gender ethnic diversities		X	X	X
Diagnostic studies- interpret clinical laboratory findings and diagnostic imaging of NMSK	X	X	X	X
Diagnosis & differential diagnosis	X	X	X	X
Case management/Referral	X	X	X	X
Chiropractic adjustment or manipulation skill, competent care	X	X	X	X
Emergency care		X		X
Case follow-up and review	X	X		X
Record keeping	X	X		X
Doctor-patient relationship/communication	X	X	X	X
Professional issues/continuing education/Sound business practice/ethical practice	X	X	X	X
Other therapeutic procedures	X	X	X	X
Public health and community interaction*	X	X	X	
Health care system interaction*		X	X	
Professional interaction*	X	X	X	
Staff and financial management*		X		
Information and technology**	X			

Indicates domains from other than CCE-Canada* CCEA** CCE-USA

Despite the differing structures, there was considerable agreement among CCEs. All stipulated that competence required the domains of history taking, physical examination, differential diagnosis, imaging, laboratory testing, chiropractic adjustment/manipulation skill, management, delivery of care and communication. Finally, competency was expected in the domains of ‘professional issues’, ‘continuing education’, ‘sound business and ethical practice’, ‘public health’, and ‘community and professional interaction’.

Despite high similarity levels, also differences were noted. The same terms or phrases describing competencies were found at the domain or subdomain level in different CCEs. For example the CCE-Canada and CCE-Aust included competence in dealing with an emergency medical situation while assessing or providing care. The ECCE and CCE-USA included it at the subdomain level. The CCE-USA did not state the need for a psychosocial assessment at the domain (metacognitive) level; rather it was presented as a component of the domain of assessment and diagnosis.

Some CCEs had unique domains. The CCE-Aust specified the area of staff and financial management and the CCE-USA required competence with information and technology.

The only notable omission was that the ECCE did not state the need for case follow up or review and did not specifically mention competent record keeping.

### Objective 3: analysis of three important domains

All components of the three selected domains of ‘assessment and diagnosis’, ‘professional ethics and jurisprudence’, and ‘intellectual and professional development’, were tabulated and presented in Table 4.

**Table 4:** Descriptions used by CCEs of the three selected representative domains of “assessment & diagnosis”, “professional jurisprudence and ethics” & “intellectual and professional development”

*Competency dimension :assessment and diagnosis*	CCE-USA	CCE-Aust	ECC-Europe	CCE-Canada
Background clinical sciences				
Understand the pathophysiology and history of NMSK conditions			X	X
Understand the signs and symptoms of NMSK conditions			X	X
Understand the prognosis of NMSK conditions			X	
Case history				
Data gathering (CCE USA)	X	X	X	X
Data recoding	X	X	X	X
Take a comprehensive problem-focused or case-appropriate history	X	X	X	X
Psychosocial factors considered in case history taking	X	X	X	X
Cultural ethnic issues considered specific to case history taking		X	X	X
Patient centred/comfort when history taking		X	X	X
History taking subcomponents specified eg chief complaint, family, past, systems review	X		X	
Practitioner behaviours describe during the process		X		X
Physical exam/assessment				
Perform an appropriate general physical exam	X	X	X	X
Perform an appropriate case appropriate/NMSK physical exam	X	X	X	X
Description of physical exam components				X
Incorporate psychosocial assessment	X	X	X	X
Incorporate subluxation/neuro-biomechanical dysfunction	X	X		X
Reliability of data/tests/ examinations considered		X		X
Patient-centered requirement, comfort, respect + psychosocial factors assessment		X	X	X
Doctor hygiene and patient safety				X
Explanation of findings to patient		X		X
Radiology – with specific requirements				
Radiological Interpretation		X		X
Radiographic technology		X		X
Laboratory tests				
General statement for requirement of utilization & interpretation competence	X	X	X	X
Risk/cost benefit analysis		X	X	X
Within scope of practice		X	X	X
Ordered based on previously obtained clinical data		X		X
Explained to patient		X	X	X
Diagnosis				
Formulate a diagnosis(es) based on information gathered-general statement	X	X	X	X
Documentation of diagnosis	X	X		X
All material considered in the diagnosis	X	X	X	X
Use diagnosis for recognition of when condition exceeds capacity/referral		X	X	X
Explanation of diagnosis to patient		X	X	X
Within the context of clinical reasoning skills/problem-solving skills	X	X	X	X
*Competency dimension : professional ethics and jurisprudence*	CCE-USA	CCE-Aust	ECC-Europe	CCE-Canada
Ethical principles & professional conduct	X	X	X	X
Patient – practitioner boundaries: physical, communication (verbal, non-verbal) emotional	X	X		X
Knowledge of health care law	X	X		X
Professional conduct with peers	X	X	X	X
Professional conduct with patients	X	X	X	X
Professional conduct with staff	X	X		X
Compliance with ethical and legal dimensions	X	X	X	
Patient records and patient billing meets state and federal law	X	X		X
Ethical business practices		X		X
Professional participation/support		X		X
Explain the importance of research participation		X		X
*Competency dimension : intellectual and professional development*	CCE-USA	CCE-Aust	ECC-Europe	CCE-Canada
Seeking and application of new knowledge	X	X	X	X
Ability to adapt to change	X	X	X	X
Critical appraise literature and apply it to clinical practice/patient care	X	X	X	X
Understanding of research methods and significance in modern health care	X	X	X	X
Provide evidence of critical thinking skills	X	X	X	X
Reflect on personal and professional learning skills	X	X	X	
Application into patient care	X	X	X	X
Demonstration of basic, social and clinical sciences sufficient to promote intellectual development and effective patient care	X			

#### First domain - assessment and diagnosis

This domain was described using a number of subarea statements. Two of the CCEs (Europe and Canada) expected that assessment and diagnosis would be underpinned by background knowledge of clinical science. They stated that this should be evidenced by an understanding of the pathophysiology, history and signs and symptoms of neuromusculoskeletal conditions. Only the CCE-Canada included prognosis of musculoskeletal conditions. The CCE-USA and CCE-Australia made no mention of clinical science competence. CCEs described “Assessment and Diagnosis” by breaking it down into smaller components. These tended to *case history, physical examination, investigations/laboratory testing/imaging, and diagnosis*.

#### Case history

All CCEs used the term “case history” when describing assessment and diagnosis. However they differed in the number of components they used to define it. The CCE-Aust used the word “*comprehensive”* to define the taking of a case history*.* The remaining CCEs stated it need not be comprehensive but could be problem focused or case appropriate. Two of the CCEs (CCE-USA and CCE-Canada) defined the components of a case history as being a chief complaint, a systems review, and family history while the others did not.

The CCE-Aust and the CCE-Canada stated the need for consideration of patient comfort (physical and psychological) and a display of empathy (verbal and non-verbal) during history taking whereas the CCE-USA and ECCE did not.

#### Physical examination

There was an expectation of a physical examination by all CCEs but the difference in the number of descriptive statements was considerable.

The ECCE simply asked for “*an appropriate physical examination for the purpose of arriving at an appropriate diagnosis”*. The CCE-USA described it as *“performing case appropriate physical examinations that include evaluations of body regions, organ systems including the spine and any subluxation/neuro-biomechanical dysfunction . . .for developing the clinical diagnosis”*. The CCE-Canada was the only other CCE to use the word subluxation in this context, defining it as being an “articular subluxation”.

The CCE-Aust and CCE-Canada were more prescriptive. They viewed this domain as comprising two components; a general physical examination (15 descriptive statements by the CCE-Aust and 16 by the CCE-Canada) and a neuro-musculoskeletal examination (6 and 14 statements, respectively). Consequently they were able to include issues such as a patient-centred approach to physical examination, the reliability and appropriateness of examination procedures and findings, and an explanation of these to the patient. Only the CCE-Canada required practitioner hygiene and patient safety considerations during a physical examination.

The ECCE and CCE-USA stated that a physical examination is to be used to formulate a diagnosis. The CCEs of Australia and Canada expanded this purpose so that the physical examination was also required to evaluate the patient’s clinical status, monitor change, and rate disability and impairment.

#### Investigations/laboratory tests/imaging

All CCEs expected competent interpretation and appropriate use of laboratory tests. Competency interpreting advanced imaging (such as MRI, CAT scans or musculo-skeletal ultrasound) was not specified by any CCE.

The CCE-USA statement with regard to diagnostic competency was: “*utilizing diagnostic studies and consultations, where appropriate, inclusive of imaging, clinical laboratory, and specialized testing procedures, to obtain objective data”.* Unlike the other CCEs it did not specify the need for consideration of risk/cost benefit when ordering laboratory tests or imaging. It also permitted a chiropractor to order a test for any condition rather than those related to neuromusculoskeletal issues. This may be a reflection of a broader scope of practice available to USA chiropractors. Further there was no requirement to explain the findings to the patient.

The ECCE stated that the chiropractor should be able to “*interpret diagnostic procedures …and their uses and limitations . .* .” whereas, again, the CCEs of Australia and Canada were more prescriptive. The CCE-Aust had 25 descriptive statements (11 for radiographic interpretation and 14 for radiographic technology) and included items such as “*radiographic data being used to confirm the accuracy of the presumptive diagnosis initially identified . . . . each radiograph is thoroughly scrutinised in an organised manner…adequate patient protection is used…exposure technique uses safety parameters*”. The CCE-Canada stated: “…*understands the principles, applications, technical and procedural elements of equipment employed in diagnostic imaging . . . . . take, process and interpret plain film radiographs with appropriate attention given to quality and safety”.*

#### Diagnosis

There was global agreement with the need for a competent chiropractor to be able to gather, document and analyse patient information, refer to others (if indicated) and arrive at a list of differential diagnoses. All CCEs recognized the need for an overarching competence in clinical reasoning/problem-solving skills. The CCE-USA was the only one that did not require communication of these findings to the patient. The ECCE made no statement on the need for appropriate or adequate documentation or clinical records.

### Domain 2: professional ethics and jurisprudence

All CCEs expected that the chiropractor should behave in an appropriate ethical and professional manner. This involved appropriate conduct/communication with peers and other health care providers.

The CCEs used different terms to describe the context and application of ethical and professional behaviour. The CCE expectations were: for this performance to occur at the highest possible levels (ECCE), that chiropractors exhibit this behaviour (CCE-Canada), that it is complied with and maintained (CCE-USA), and that graduates are expected to be aware of professionalism and display it (CCE-Aust).

Some differences were noted. The CCE-Canada stipulated that ethical practice should include ethical business practices while the CCE-Aust expected subscription to the professions code of ethics and adherence to the legal requirements of conducting a practice. The CCE-USA specified ethical business standards as including the meeting of legal requirements for patient records and billing codes, and professional conduct with staff in accordance with established policies. All, except the ECCE, stated that they expected professional conduct with staff. Finally, neither the ECCE nor the CCE-Aust mentioned the need for patient-practitioner boundaries whereas the others did.

The CCE-Canada and the CCE-Aust required the competent practitioner to support and participate in professional activities, although this was not defined. They both also expected active knowledge of research and its use for the profession. The CCE-USA and the ECCE wanted the individual practitioner to have knowledge of research methodologies and the ability to critically appraise scientific literature and incorporate this into patient care. The ECCE additionally stated the need for contribution to the generation of knowledge and the education of junior colleagues.

### Domain 3: intellectual and professional development

All CCEs described the competent practitioner as one who seeks new knowledge, critically evaluates it and would apply new knowledge to patient care over the duration of their professional lives. Further, all CCEs expected an understanding of research methods and its significance in modern health care. Additionally, all CCEs expected competency in critical thinking to evaluate current and new knowledge. The term “evidence-based” was generally absent, only used by the ECCE.

Some minor differences were noted. The CCE-USA alone expected an ability to reflect on personal and professional learning skills. The CCE-Aust specified activities for professional scientific development such as the ability to give a case presentation with an adequate review of the literature.

## Discussion

This review appears to be the first systematic approach to investigate similarities and differences between definitions of competency and graduate competency standards of the various CCEs. In general these definitions were more similar than dissimilar. There was considerable agreement in the choice of definitions for competency and the content at the domain level describing it. However, there was discernible variation in the degree of prescriptiveness of the CCEs when describing competency standards. Meaningful differences became increasingly evident when comparing the component lists describing the domains. In a worst case scenario such differences could result in incompetent emergency care, inadequate case history and physical examinations, inappropriate radiological utilization, and poor comprehension of patient-practitioner boundaries.

### Definitions of competence

CCEs were found to be similar to medical councils on education as they both included “*knowledge*” and “*skills*” as important elements when defining competence [5]. Unlike medical councils on education, all CCEs included “attitudes” but only one included “abilities”. While this may be due to differences in the understanding of the words used by CCEs, it speaks to the need for agreement between CCEs on the definition of common words used in their documentation.

However, a health practitioner’s attitudes and knowledge may not reflect his/her actual behaviour [22]. By including the element “abilities” in definitions, it would be possible to measure a greater range of behaviours, which in turn would make them more professionally specific and useful for assessment purposes [8]. The CCEs were dissimilar with respect to the additional elements they included in their definitions when compared to medical councils on education definitions of competence. There were only two; psychomotor skills and problem solving abilities. The medical education literature recognises a much broader understanding of additional elements [5]. This includes, but is not limited to, communication, assessment, collaboration, and advocacy. These, along with problem solving are seen as important underlying factors for medical graduate competency lists [23]. Also chiropractic practice requires these dimensions as well as manual skills and abilities. Consideration on how to address all these dimensions in CCE definitions would add to their clinical usability.

The purposes of the CCE definitions were either to monitor students’ progress or to determine if they were ready to serve as a chiropractor. Assessing a student at a fixed point in time, such as at a competency exam before graduation, may be a poor moment for monitoring progress. The determination of a student’s competence development also requires the capacity to inform the educators how effective a curriculum is in producing a graduate with desired qualities for the profession [24]. Non chiropractic examples, such as reported in the recent review of Australian health-force competency-based education, included the basic elements of knowledge, skills, decision making attributes and values when assessing the students’ preparedness for graduation. However these were applied to “*performing tasks safely and effectively in a specific health workforce role*” [25]. A definition such as this, with safety as a central issue, may create opportunities for more specific and effective graduate competencies and improve its applicability for chiropractic students and graduates. Alternatively, one specific definition may be required for monitoring students’ progress and another for determining whether or not they are competent for graduation and solo practice.

In sum, knowledge, skills, and attitudes are common components of definitions of chiropractic competence. Nevertheless, further work is needed to clarify other useful profession-specific dimensions such as the types of abilities and skills required and the time at which they should be assessed.

### Domain analysis

Despite the considerable variability in the number of domains, a high degree of content similarity was found at this level between the CCEs.

CCE regulations were similar in the approach they took to constructing domains. They tended to fragment the clinical encounter chronologically i.e., case history, assessment, diagnosis, and case management. Recent medical trends have moved away from this thinking and have constructed domains that encompass overarching aspects of practice. For example, the Canadian system of CanMEDS describes these in terms of roles such as medical expert, communicator, collaborator, scholar, health advocate and professional [26]. The American medical system, as exemplified by the Accreditation Council for Graduate Medical Education, ACGME, [27], utilises the domains of patient care, medical knowledge, practice based learning and improvement, professionalism and interpersonal skills, and communication [27]. These approaches have been well funded, developed by eminent physicians and academics, and are built on extensive clinical experience and the highest quality evidence. As such they deserve serious consideration for their applicability and relevance also to chiropractic education. This would allow chiropractic educators access to researched and validated medical education changes based on this structure which will in turn improve educational outcomes. Indeed, past research has suggested that European chiropractic students and practitioners consider the 7 domains of CanMEDS as being important and highly applicable to chiropractic graduate training [28].

### Component analysis of three representative domains

The domain structures among CCEs were sufficiently similar to allow identification for comparison purposes. However, it was apparent that CCEs had used differing levels of prescriptiveness, when describing these.

The first difference noted was that two CCEs included an expectation of ‘fundamental knowledge of pathophysiology’ and the remaining did not. One possible explanation is that it could be assumed that there would be enough knowledge underpinning other components and that it does not require stipulation. For example, pathophysiology is required to competently construct a differential diagnosis. Its absence would be indicated by inadequate diagnostic skills, which is a stated expectation among all CCEs. Nonetheless consideration should be given to the clear stipulation of the knowledge expected of chiropractic graduates relevant to CCE competencies. This would improve the educational institutions’ ability to understand and meet the CCE graduate expectations.

In general, there were demonstrated differences in the prescriptive approaches taken by the CCEs. This was exemplified in the contrasting standards for radiographic imaging of the CCE-Canada and CCE-USA. Historically, chiropractic treatment systems have been documented to overuse radiography [29–31]. Reasons for this have included diagnostic uncertainty, fears of missing contraindications to manipulation, financial gain, routine screenings, and biomechanical considerations [29, 32]. The CCE-USA radiographic/imaging requirements were minimal and did not possess the detail to specifically address many of the reasons for radiography overuse. However, the CCE-Canada imaging related statements contained more detail thus specifically addressing appropriate levels of use. This difference between the two CCEs may provide a possible mechanism for the existence of practitioner variations found in X-ray utilisation in the Canadian practice profiles, as referred to in our introduction [13]. Further, all CCEs need to include more competency expectations for contemporary imaging modalities.

While all CCEs may be similar in the use of certain words or terms they were sometimes dissimilar in the way they understood that word and in how they intended it to be applied to clinical practice. For example, all of the CCEs utilized the term “physical examination”. The descriptive terms CCEs used to describe it ranged from performing an “appropriate physical examination .….to arrive at a diagnosis” through to a more prescriptive approach of conducting a complete physical and targeted neuro-musculoskeletal examination. The more prescriptive approach specified the exam procedures and their function as a means of developing a diagnosis, rating disability and impairment, and for monitoring patient change. The dissimilar approach was also seen with the various terms used to describe the focus of the examination such as “*subluxations”* “*articular subluxation”* and *“neurologic and orthopaedic dysfunction”.* Recent research has suggested that 63 % of medical errors were a result of failure to perform a physical examination [33]. As a consequence of physical examination inadequacy, 76 % of cases included a missed or delayed diagnosis and 18 % received incorrect treatment. This suggests that CCEs should consider the evidence for a more prescriptive approach to the components of a physical examination to reduce the possibility of errors. Further there is a need for a clear understanding of how the physical examination relates to chiropractic practice. The terms currently used, such as “subluxation” are not reproducible clinically diagnosable entities [34]. This is further complicated by the general difficulty in arriving at a diagnosis for commonly encountered chiropractic conditions, such as low back pain [35]. An evidence-based approach to these clinical uncertainties has been proffered and warrants consideration [36].

The domain and the descriptive statements for *Intellectual and professional development* were very similar among CCEs. Competent graduates were expected to be lifelong learners who could critically evaluate and apply new and existing knowledge. Only one CCE used the widely accepted and commonly used medical education term of an evidence-based approach, when describing this domain [37]. There is substantive research surrounding this approach to learning and professional development [38]. Recent research found that 46 % of USA chiropractors did not take evidence-based practice into account when making clinical decisions [39]. However, this study found that 85 % of these respondents were interested in improving their skills necessary to incorporate it into their practices. This would suggest that further emphasis by CCEs may be required to continue to promote evidence-based practice.

Its use in chiropractic competency lists may be part of that process. Further, using a common language may improve communication between health educational bodies and integration within the health field [40]. Consequently, consideration should be given to wider use of the term “evidence-based” in graduate competencies among CCEs.

While all CCEs were similar in the recognition of a domain for ethical and professional behaviour, they were dissimilar in the description of its expression in practice. They varied from expecting it to occur at the “highest possible levels” through to being “aware of it”. Ethics education in North America and Canadian Chiropractic colleges appears to be very diverse with variable content and no common reference reading materials [41]. Of concern is that variations in chiropractic course content may result in some important ethical areas being omitted. For example, this review found that only two of the four councils on chiropractic education made specific reference to the need for patient practitioner boundaries. Several solutions have been suggested. One is a broader based and more congruent undergraduate ethics curriculum [41]. This would also have the additional benefit of increasing the trend of chiropractic integration into mainstream health care settings [41]. Another recommendation could be to increase chiropractic education and training in the area of practitioner behaviour [42]. The presence of these recommended changes in competency standards would give chiropractic educational programs guidance on course content and warrants serious consideration for inclusion.

Differences in the wording of ethical business practice description lists were noted among CCEs. These varied from a general expectation of conducting ethical and legal business standards to the meeting of the legal requirements for patient records and billing codes. There are known issues in chiropractic practice such as unsubstantiated claims in patient brochures [43], wellness practice based on “vitalism” tenets [44], the sale of “good health” products [45], and anti-immunization views [12] which are not addressed by the current descriptive lists by all CCEs. However the descriptive list from *intellectual development* which expects the practitioner to know and apply current knowledge, if enforced, should theoretically restrict these behaviours.

Of course, other mechanisms may account for these aberrant practice behaviours that develop after graduation other than insufficiently detailed CCE competencies. While one explanation could be that CCEs in certain geographical locations may not be enforcing these standards. Other factors may also be post-graduation practice expectations from work colleagues, practitioner personality types, and adoption of a chiropractic technique which are at odds with these regulatory competencies.

In sum, CCEs take varying approaches to the task of constructing graduate entry-level standards. A less prescriptive approach is typified by general guidelines and discretion appears to be given to the educational institutions to interpret and implement them. This creates the capacity for innovative teaching and practice. The opposite may be viewed as a more directive or prescriptive approach. While this reduces the potential for innovative teaching styles, it also reduces the capacity for deviation and potentially irregular practice profiles.

### Recommendations

This review has sought to identify similarities and differences between CCEs internationally in their definitions of competence and graduate entry level competencies. This has led to the identification of a number of issues and, based on these, we make a number of recommendations that are summarised in Table 5. If these recommendations were adopted then outcomes such as a uniform high standard of practitioners who are evidence-based and lifelong learners is likely across all CCE-controlled regions. This would ensure and safeguard the international trust in practitioners’ ability to deliver ethical, safe and quality care across international borders.

**Table 5:** Summary table of recommendations

	Recommendations in relation to competencies	Justifications
1	An internationally uniform definition of competence for chiropractic education and assessment is required.	There is increasing global workforce movement and there is evidence of variations in international standards. Common standards would ensure and safeguard patient safety and care and be good for global workforce standardization
	This may require agreement from all CCEs on the definition of common words and terms used in their documentation.	
2	There should be separate definitions of competence at different stages of the course work; separating the undergraduate’s progress from readiness to graduate.	Chiropractic educators are better equipped to monitor and assess a student’s progress toward detailed graduating standards.
3	“A*bilities”* and *“other categories”* should be included in the definition of competence and their meanings clarified among CCEs.	This would create a clearer understanding of the required standards to be assessed and achieved by chiropractic educators.
	Recommendations in relation to domains	
4	A clarification of the use of the terms and words used to describe the domains of competency should be undertaken so there is an established understanding of their meaning among CCEs.	High levels of descriptions reduce the capacity for ambiguity as they clearly state the expected behaviours and standards of graduates.
5	Common domains of competency need to be created for chiropractic education. These domains should reflect not only practitioner behaviours but also qualities and roles. Consideration should be given to recent examples such as CanMEDS [46] and the ACGME [47]	Adoption of these structures would also improve the likelihood of mainstream integration.
6.	Appropriate descriptive statements should be found that adequately define the domains, sub-domains and their components. These should be sufficiently prescriptive and unambiguous to establish high standards of practice and reduce the possibility of undesirable practice profiles. E.g., radiology competencies, physical examination, and pathophysiology expectations.	CCEs should consider the evidence for a more prescriptive approach to component descriptive statements that would set clearly defined quality graduation standards for educators to achieve and CCEs to enforce.
7	The term “evidence-based” should be used for improved research and knowledge application, such as patient safety and treatment improvements from other mainstream medical disciplines. Further it would facilitate communication and integration within the broader health field. Content taught should be required to be done in the context of the evidence that underpins it.	The adoption of an evidence-based approach would help facilitate integration into mainstream health care.
8	Increased description of ethical and professional practice and practitioner behaviours which are consistent across all CCEs.	Clarity would ensure and safeguard high professional standards.
9	Imaging competencies need to include contemporary modalities such as MRI, CT and diagnostic ultrasound	Health care technology is constantly changing and chiropractic education should keep pace with these changes, so that patients benefit from access to these emerging imaging technologies.
10	CCEs should guide and fund research into accreditation matters: suggested areas include, but not limited to;	This will develop, inform and improve regulatory standards
10 (a).	A study comparing CCEs’ levels of enforcement of competency standards.	Identifying the opportunities for improving enforcement of standards may result in a uniform quality international standard of patient care and safety of practice.
10 (b).	A study of factors that may be at odds with competency standards.	Identification of these factors may provide opportunities and mechanisms for chiropractic educators to improve competency levels.
10 (c).	A study trialling interventions targeted at improving identified unwanted practitioner profiles which may alter practice behaviours.	This would improve the quality of patient care and safety

### Methods/Considerations

A potential weakness of this study is the subjective nature of the interpretation of the structure for the analysis of the domain and component statements. Our choice of domains may differ from others. For example we selected for convenience the 14 domains of CCE-Canada as a comparative analyses structure. There may be other possible constructions for analysis which may impact on the differences and similarities observed.

The strengths of this review are that it did take a systematic approach and that the two investigators extracted the information with a high level of agreement. Further, all available information was covered and analysed.

## Conclusions

This systematic review investigated and identified similarities and differences between the various CCEs in their definitions of graduate competency and the educational competencies they have adopted. The main similarities were found in relation to the structure and terms describing the domain level of competencies. Differences were noted in the interpretation, of those terms. These differences were more pronounced at the component descriptive level. Consequently, a series of recommendations were made. The adoption of these has the potential to create a homogenised, internationally consistent, and high quality set of graduating standards.

Variations in international standards of competency may also be influenced by CCEs differences in enforcement standards or accreditation criteria. This suggests the need for studies comparing similarities and differences of chiropractic college self-evaluation reports and rejoinders to CCE responses, CCE accreditation/inspection team reports, and final reports of findings.
